# Prognostic determinants and functional role of PIK3C2G in stage IIb-IIIa lung adenocarcinoma: insights from clinical and molecular analyses

**DOI:** 10.3389/fonc.2024.1473437

**Published:** 2025-01-30

**Authors:** Chao Gao, Jiaqi Zhang, Xin Du, Xuehan Gao, Xiayao Diao, Ke Zhao, Yeye Chen, Shanqing Li

**Affiliations:** Department of Thoracic Surgery, Peking Union Medical College Hospital, Chinese Academy of Medical Sciences and Peking Union Medical College, Beijing, China

**Keywords:** lung adenocarcinoma, stage IIb and IIIa, whole-exome sequencing, PIK3C2G, targeted therapy

## Abstract

**Background:**

To investigate the prognostic factors for stage IIb and IIIa lung adenocarcinoma following radical surgery and to explore the molecular mechanisms underlying these prognostic markers, focusing on the role of PIK3C2G.

**Methods:**

A retrospective analysis of patients with stage IIb or IIIa lung adenocarcinoma who underwent radical surgery between January 2017 and June 2023 was conducted. Baseline clinical and pathological data, surgical methods, and postoperative treatments were analyzed to assess overall survival (OS). Univariate and multivariate Cox regression analyses were conducted to identify prognostic factors. Whole-exome sequencing (WES) was performed on a subset of the patients with preserved tumor tissues and no matched targeted therapies to identify high-frequency mutated genes. Functional experiments in A549 lung adenocarcinoma cells were performed to evaluate the role of the significant genes in tumor progression through cell proliferation, migration, invasion, apoptosis, and cell cycle assays.

**Results:**

The survival analysis of 877 stage IIb and IIIa lung adenocarcinoma cases revealed significant differences in clinical characteristics and outcomes. Stage IIb patients had a median OS of 58 months compared to 37 months for stage IIIa, with 5-year OS rates of 46.9% and 30.5%, respectively. Univariate and multivariate Cox regression identified pathological stage, number of positive lymph nodes, age, and targeted therapy as independent prognostic factors. WES of 184 patients with no matched targeted therapies revealed high-frequency mutations in genes such as TP53 and PIK3C2G, with the latter emerging as the most significant prognostic marker. Functional assays demonstrated that the knockdown of PIK3C2G in A549 cells significantly reduced proliferation, migration and invasion while promoting apoptosis and disrupting cell cycle progression.

**Conclusion:**

PIK3C2G was identified as a significant prognostic marker in stage IIb and IIIa lung adenocarcinoma, with functional data supporting its therapeutic potential. Taken together, this study integrates clinical and molecular findings, which could be used as a reference to guide personalized treatment strategies.

## Introduction

Lung cancer remains the leading cause of cancer-related mortality worldwide, with approximately 1.8 million deaths annually, according to GLOBOCAN 2018 data from the International Agency for Research on Cancer (IARC) (1). The prognosis of lung cancer is closely tied to its stage at diagnosis, with five-year survival rates ranging from 77% to 92% for stage Ia non-small cell lung cancer (NSCLC) but plummeting to just 9% for stage IV disease (2). Treatment guidelines from the National Comprehensive Cancer Network (NCCN) recommend surgery as the primary intervention for stage I and IIa NSCLC, while systemic therapy is preferred for stage IIIb and IV cases (3). However, optimal treatment strategies for stage IIb and IIIa NSCLC remain a subject of debate. Key issues include the role of surgery, the risks and benefits of neoadjuvant therapy, the selection and necessity of adjuvant therapy, and the individualized application of targeted and immunotherapy.

Surgical management of stage IIb and IIIa NSCLC is particularly complex. In stage IIIa disease, local tumor invasion into adjacent tissues or lymph nodes often necessitates multimodal treatment approaches, including combinations of surgery, chemotherapy, and radiotherapy. Neoadjuvant therapy, designed to reduce tumor size before surgery, can improve resectability but carries risks such as surgical delays and treatment-related side effects. Similarly, postoperative adjuvant therapy aims to reduce recurrence risk, but optimal regimens depend on patient characteristics and remain a subject of clinical debate. Furthermore, advancements in targeted and immunotherapy have opened new avenues for individualized treatment based on tumor-specific genetic markers. However, their efficacy, appropriate application, and integration into multimodal treatment strategies for stage IIb and IIIa NSCLC are not yet fully established (4–6). Thus, comprehensive research is needed to refine these treatment strategies, improve survival rates, and guide future innovations in care.

NSCLC accounts for approximately 85% of all lung cancer cases, with adenocarcinoma being the most prevalent subtype, representing 40% to 50% of cases. While radiotherapy and chemotherapy remain essential components of treatment, targeted and immunotherapies are increasingly used, especially for adenocarcinoma. Despite these, surgical resection remains the cornerstone of treatment for potential curability, and therefore, understanding the factors that influence prognosis following surgical resection is an important area of clinical research.

This study addresses the pressing need for improving outcomes in stage IIb and IIIa lung adenocarcinoma patients through a comprehensive approach integrating clinical, molecular, and cellular analyses. By retrospectively analyzing survival data of 877 patients who underwent radical surgery, the study identifies critical clinical and pathological prognostic factors, such as targeted therapy, tumor stage, and lymph node involvement. Furthermore, through whole-exome sequencing, novel insights into the genetic landscape of these patients were uncovered, highlighting PIK3C2G as a potential therapeutic target. Functional validation experiments in lung cancer cell models confirmed its role in tumor progression. The findings bridge clinical outcomes with molecular mechanisms, offering a foundation for personalized treatment strategies and paving the way for future therapeutic advancements in lung cancer care.

## Methods

### Patient cohort and study design

First, we retrospectively retrieved the data of patients who underwent radical lung cancer surgery in the Department of Thoracic Surgery of Peking Union Medical College Hospital (Beijing, China) between January 2017 and June 2023. The inclusion criteria for study analysis were as follows: (1) pathological confirmation of lung adenocarcinoma; (2) postoperative pathology confirming stage IIb or IIIa lung cancer according to the AJCC 8th edition TNM classification; (3) availability of complete clinical and follow-up data; and (4) completion of radical surgery. For this study, radical surgery was defined as complete lobectomy of the tumor-containing lung lobe, resection of other invaded lobes or structures without residual tumor, and systematic lymph node dissection of at least three intrapulmonary and three mediastinal lymph node groups, including subcarinal nodes.

Cases were excluded if they: (1) received preoperative neoadjuvant therapy; (2) underwent palliative, compromised, or residual positive-margin surgeries; (3) had multiple lesions that could not be clearly distinguished between intrapulmonary metastases and multiple primary lesions; and (4) died within three months after surgery due to complications. All patients were reclassified and restaged according to the NCCN Clinical Practice Guidelines for NSCLC.

This study was conducted in accordance with the Declaration of Helsinki (revised 2013) and approved by the Institutional Review Board of Peking Union Medical College Hospital. Informed consents were obtained from all patients before surgery for the anonymous use of their data for scientific purposes.

### Follow-up

Postoperative follow-ups were conducted up to December 31, 2023, to assess their overall survival (OS), which was defined as the time from surgery to death or the date of the last known follow-up. Patients who were still alive at the final follow-up date were censored.

### Sample collection for whole-exome sequencing

Tumor samples of the postoperative lung adenocarcinoma patients who met the following inclusion criteria were retrieved and used for further analysis: (1) absence of matched targeted therapy postoperatively; (2) preservation of tumor tissue and adjacent normal tissue during surgery; and (3) sufficient tumor specimen quality for sequencing. The tumor samples, approximately the 0.5-1 cm in size, were collected within 30 minutes of surgery and immediately frozen in liquid nitrogen. Then, the samples were stored in ultra-low temperature freezers at -80°C.

### Whole-exome sequencing protocol

DNA from the collected tumor and adjacent tissue samples were extracted and processed. The main instruments used included the Agilent 2100 Bioanalyzer (Agilent Technologies, Santa Clara, United States), Bio-Rad CFX96 Real-Time PCR System (Bio-Rad Laboratories, Hercules, United States), Covaris M220 Ultrasonicator (Covaris, Inc., Woburn, United States), Dynal DynaMag-2 Magnetic Rack (Thermo Fisher Scientific, Waltham, United States), Eppendorf 5810R Centrifuge (Eppendorf AG, Hamburg, Germany), Illumina HiSeq 2500 Sequencing Platform (Illumina, Inc., San Diego, United States), NanoPhotometer Spectrophotometer (Implen GmbH, Munich, Germany), Qubit 3.0 Fluorometer(Thermo Fisher Scientific, Waltham, United States). Initially, the tissue samples preserved at -80°C were prepared by grinding them into fine powder using a mortar pre-cooled with liquid nitrogen. The powdered tissues were then transferred into 1.5 mL centrifuge tubes containing separation buffer. In parallel, 2 mL of EDTA-anticoagulated peripheral blood was processed by lysing red blood cells with distilled water, followed by centrifugation to isolate white cell precipitates. This lysis process was repeated to ensure purity.

DNA samples were extracted from the tissues and white blood cells, and their quality and concentration were assessed using a NanoPhotometer spectrophotometer and agarose gel electrophoresis. DNA samples were included if they exhibited no or minimal degradation and were in a concentration of at least 50 ng/μL. High-quality DNA was then fragmented into segments of 100–300 base pairs (bp) using a Covaris M220 ultrasonic device, then subjected to end-repair and A-tailing processes to prepare them for adapter ligation. Adapter-ligated fragments were purified using magnetic bead-based methods.

The purified DNA fragments underwent polymerase chain reaction (PCR) amplification to create pre-libraries, which were subsequently enriched for exonic regions using the SeqCap EZ MedExome Enrichment Kit. The resulting libraries were quantified using the Qubit 3.0 fluorometer and sequenced on the Illumina HiSeq 2500 platform.

Raw sequencing data were assessed for quality to remove low-quality reads. The processed reads were aligned to the reference human genome using the Burrows-Wheeler Aligner (BWA) to ensure accurate mapping. Variants, including single nucleotide variants (SNVs) and insertions/deletions (Indels), were detected and their quality values were calculated using the Genome Analysis Toolkit (GATK). Additionally, copy number variations (CNVs) were identified using CONTRA software, and MutSigCV was employed to identify significantly mutated genes. Finally, the mutation data were visualized and interpreted using the “maftools” R package.

### Functional analysis of PIK3C2G in A549 cells

#### Cell culture

A549 cells, a cell line derived from human alveolar (pneumocyte type II) adenocarcinoma of a single Caucasian male, were rapidly thawed in a 37°C water bath while gently shaking to ensure uniform and expedited thawing. Upon complete thawing, the cell suspension was centrifuged at 1000 rpm for 5 minutes to separate the cells from the cryoprotectant. Then, the supernatant was carefully discarded to avoid disturbing the cell pellet. The cell pellet was resuspended in 1 mL of complete culture medium comprising DMEM supplemented with 10% fetal bovine serum (FBS) and 1% penicillin-streptomycin, gently mixed and transferred to a 10 cm culture dish containing 10 mL of the complete culture medium. The culture dish was incubated overnight at 37°C in a humidified atmosphere containing 5% CO_2_ to allow cell adhesion. The following day, the culture medium was replaced to remove non-adherent cells and accumulated metabolic waste. The medium was subsequently replaced every other day to maintain optimal culture conditions. On the third day of incubation, the cells were subcultured using a 1:3 split ratio to preserve exponential growth and genetic stability to ensure they remained in a healthy and replicative state for subsequent experiments.

#### Cell transfection

The growth state of the A549 cells was assessed under an inverted microscope to ensure optimal conditions for transfection. The cells were then digested with 0.25% trypsin-EDTA to generate a single-cell suspension, which was centrifuged at 1000 rpm for 5 minutes, and the supernatant was discarded. The resulting cell pellet was resuspended in fresh complete culture medium, and the cells were counted using a hemocytometer. The cell concentration was adjusted to 2 mL per well, and the suspension was seeded into 6-well plates. The plates were incubated at 37°C in a humidified atmosphere with 5% CO_2_ for 24 hours to allow the cells to adhere and reach the desired density for transfection. For gene silencing experiments, 1 μg of siRNA targeting human LTK (NM_002344) and PIK3C2G (NM_004570) was added per well using Lipofectamine 2000, following the manufacturer’s protocol. The transfection mixtures were gently mixed and incubated for the specified time to ensure efficient siRNA delivery. The cells were then maintained under standard culture conditions for further downstream analysis.

### RNA extraction

Total RNA was extracted from the transfected A549 cells using the Trizol reagent, and the lysate was centrifuged at 2000 rpm for 5 minutes to remove impurities. The cell pellet was resuspended in 1 mL of Trizol, thoroughly mixed, and left at room temperature for 5 minutes to complete lysis. To separate the aqueous phase, 200 μL of chloroform was added to the lysate, which was then vigorously shaken for 15 seconds and allowed to stand for 10 minutes. The mixture was centrifuged at 12000 rpm for 15 minutes at 4°C, and the upper aqueous phase containing RNA was transferred to a new tube. RNA was precipitated by adding an equal volume of isopropanol, mixed, and incubated at 4°C for 10 minutes. After centrifugation at 12000 rpm for 12 minutes at 4°C, the RNA pellet was washed with 75% ethanol, air-dried, and dissolved in 50 μL of RNase-free water. The concentration and purity of the extracted RNA were assessed using spectrophotometric methods.

### RNA reverse transcription

Complementary DNA (cDNA) was synthesized from the extracted RNA using the ReverTra Ace qPCR RT Kit. A reaction mixture of 16 μL, including 4 μL of 4× DNA Master Mix with gDNA Remover, 0.8 μg of RNA template, and nuclease-free water, was incubated at 37°C for 5 minutes. Then, 4 μL of 5× RT Master Mix II was added, and the reaction was conducted at 37°C for 15 minutes, followed by 50°C for 5 minutes and 98°C for 5 minutes. The resulting cDNA was diluted with 360 μL of double-distilled water (ddH_2_O) and stored at -20°C for subsequent experiments.

### Real-time quantitative PCR

RT-qPCR was performed to quantify gene expression levels using an SYBR Master Mixture. Each 20 μL reaction contained 10 μL of TransStart Tip Green qPCR SuperMix, 2 μL of diluted cDNA, 2.4 μL of synthesized primers, and nuclease-free water. The qPCR conditions included an initial activation step at 95°C for 3 minutes, followed by 40 cycles of 95°C for 15 seconds, 60°C for 30 seconds, and 72°C for 30 seconds. The data were analyzed using the 7500 Fast DX Real-Time PCR System, and relative expression levels were calculated. The primer sequences used for RT-qPCR are detailed in **Supplementary Table S1**.

### Cell migration and invasion assays

Transwell assays were performed to evaluate cell migration and invasion capabilities. Briefly, the transfected cells were divided into four groups: blank control, siCtrl (non-targeting siRNA), siLTK, and siPIK3C2G. The cells were digested, centrifuged, and resuspended in PBS. The cell concentration was adjusted to 1.5×10^6^ cells/mL, and 200 μL of the cell suspension was seeded into the upper chamber of a Transwell insert. For invasion assays, the upper chamber was coated with Matrigel prior to cell seeding. After 24 hours of incubation at 37°C, the cells that had migrated or invaded through the membrane were fixed with methanol, stained with crystal violet, and imaged at 100× magnification.

### Cell cycle analysis

The transfected cells were divided into the same four groups, then digested, centrifuged, and fixed in 70% ethanol at 4°C. Next, they were stained with propidium iodide (PI) and treated with RNase A solution to remove RNA interference. After incubation at 37°C in the dark for 30 minutes, the samples were filtered through a 400-mesh filter and analyzed using a flow cytometer to determine the distribution of cells in different cell cycle phases.

### Apoptosis analysis

The cells from each group were washed with PBS and resuspended in 1× binding buffer. Fluorochrome-conjugated Annexin V was added to the cells, which were incubated at room temperature for a specified time. PI was then added to distinguish early and late apoptotic cells. The samples were analyzed using a flow cytometer within 4 hours of staining.

### Statistical analysis

Statistical analyses were conducted using SPSS Statistics 27 (IBM) and R software (version 4.3.3) to evaluate the factors influencing overall survival (OS) and other outcomes. Baseline characteristics and pathological features, including gender, age, cancer stage, number of positive lymph nodes, air dissemination, vascular invasion, and surgical method, were stratified to assess their associations with survival outcomes. The effects of postoperative adjuvant therapies, such as chemotherapy, targeted therapy, radiotherapy, and immunotherapy, were also evaluated.

To identify factors associated with OS, univariate Cox proportional hazards regression was performed. Variables with a p-value < 0.1 in the univariate analysis were further evaluated using a multivariate Cox proportional hazards model, with statistical significance defined as p < 0.05. Continuous variables, such as age and the number of lymph node metastases, were dichotomized at their median values for subgroup analyses. Additional statistical methods included unpaired t-tests for continuous variables and Kolmogorov-Smirnov tests for categorical variables to examine differences between groups.

Data from functional and sequencing experiments were analyzed separately using R software. Lasso regression was used to identify optimal gene correlations. Kaplan-Meier survival curves were generated to visually represent OS, and the log-rank test was used to evaluate differences in survival distributions among groups. Prognostic factors were further validated using univariate and multivariate Cox regression models. P < 0.05 was considered statistically significant.

## Results

### Survival analysis of stage IIb and IIIa lung adenocarcinoma

Data analysis revealed distinct characteristics and outcomes between stage IIb and stage IIIa lung adenocarcinoma cases. As shown in **Table 1**, stage IIb accounted for 37.1% (325 cases), while stage IIIa comprised 62.9% (552 cases). The median age was 61 years (range 27–82) for stage IIb and 60 years (range 24 - 84) for stage IIIa. Gender distribution was similar in stage IIb (female:male ratio, 161:164), whereas stage IIIa had more male patients (301:251).

**Table 1:** The demographic and clinicopathological data of the total patients.

Characteristics	IIb/%	IIIa/%	Total/%
**Cases**	325(37.1)	552(62.9)	877
**Median Age (range)**	61(27-82)	60(24-84)	60(24-84)
**Females:Males**	161:164	301:251	462:415
**Number of Lymph Node Metastasis Sites**			
1	248(76.3)	158(28.6)	406(46.3)
2	67(20.6)	170(30.8)	237(27.0)
3	9(2.8)	102(18.5)	111(12.7)
4	1(0.3)	67(12.1)	68(7.8)
5	0	33(6.0)	33(3.8)
6	0	11(2.0)	11(1.3)
7 or more	0	11(2.0)	11(1.3)
**Air dissemination**	52(16.0)	193(35.0)	245(27.9)
**Vascular invasion**	36(11.1)	167(30.2)	203(23.1)
**Surgical Approach**			
Open	21(6.5)	41(7.4)	62(7.1)
VATS	304(93.5)	511(92.6)	815(92.9)
**Surgical Method**			
Lobectomy	306(94.2)	526(95.3)	832(94.9)
Sleeve Lobectomy	6(1.8)	5(0.9)	11(1.3)
Bilobectomy	12(3.7)	19(3.4)	31(3.5)
Pneumonectomy	1(0.3)	2(0.3)	3(0.3)
**Hypertension**	105(32.3)	210(38.0)	316(36.0)
**Diabetes**	76(23.4)	148(26.8)	224(25.5)
**Coronary Disease**	64(19.7)	125(22.6)	189(21.6)
**Smoking History**	67(20.6)	141(25.5)	208(23.7)

The bold values represent the percentage distribution of each characteristic within the respective clinical stage (IIb or IIIa). The total percentage of cases across both stages is also provided.

The median number of lymph node metastasis sites was two. In stage IIb, 76.3% of patients (248 cases) had only one metastasis site, whereas in stage IIIa, this proportion decreased to 28.6% (158 cases). The number of patients increased proportionally with the number of metastasis sites in stage IIIa. Air dissemination was observed in 16.0% (52 cases) of stage IIb and 35.0% (193 cases) of stage IIIa patients, while vascular invasion was detected in 11.1% (36 cases) and 30.2% (167 cases), respectively. Most patients underwent video-assisted thoracic surgery (VATS), accounting for 93.5% (304 cases) in stage IIb and 92.6% (511 cases) in stage IIIa. Open surgery was less common, performed in 6.5% (21 cases) of stage IIb and 7.4% (41 cases) of stage IIIa patients. Lobectomy was the predominant surgical method, performed in 94.2% (306 cases) of stage IIb and 95.3% (526 cases) of stage IIIa cases.

### Postoperative adjuvant treatment

**Table 2** provides an overview of the postoperative adjuvant treatment regimens. Among stage IIb patients, 44 received no postoperative treatment compared to 38 in stage IIIa, primarily due to advanced age, comorbidities, or refusal. Postoperative chemotherapy was administered to 98 stage IIb and 118 stage IIIa patients. Single-agent targeted therapy was more prevalent in stage IIIa (149 cases) than in stage IIb (86 cases). Combination regimens, such as chemotherapy plus targeted therapy or radiotherapy, were more frequently utilized in stage IIIa patients, reflecting a trend toward more aggressive treatment strategies.

**Table 2:** Postoperative adjuvant therapy of the total patients.

Postoperative adjuvant therapy	IIb(%)	IIIa(%)	Total(%)
None	44(13.5)	38(6.9)	82(9.3)
Only C	98(30.2)	118(21.4)	216(24.6)
Only T	86(26.5)	149(27.0)	235(26.8)
Only R	2(0.6)	2(0.4)	4(0.5)
Only I	1(0.3)	8(1.4)	9(1.0)
C and T	38(11.7)	64(11.6)	102(11.6)
C and R	17(5.2)	56(10.1)	73(8.3)
C and I	17(5.2)	44(8.0)	61(7.0)
T and R	2(0.6)	10(1.8)	12(1.3)
T and I	1(0.3)	3(0.5)	4(0.5)
R and I	0	0	0
C,T and R	10(3.1)	35(6.3)	45(5.1)
C,T and I	5(1.5)	6(1.1)	11(1.3)
C,R and I	2(0.6)	13(2.4)	15(1.7)
T,R and I	0	3(0.5)	3(0.3)
C,T,R and I	2(0.6)	3(0.5)	5(0.6)

None, no therapy; C, Chemotherapy; T, Targeted therapy; R, Radiotherapy; I, Immunotherapy.

### Survival outcomes and prognostic factors

The median follow-up time for all patients was 29.5 months (range 6.1–86.2 months), with a median survival time of 45.7 months. The 1-year, 3-year, and 5-year OS rates for stage IIb were 96.4%, 70.4%, and 46.9%, respectively, whereas for stage IIIa, these rates were 90.3%, 52.0%, and 30.5%. The median OS was 58 months for stage IIb and 37 months for stage IIIa, and log-rank test revealed significant difference in survival distributions between these two stages (p < 0.001).

**Figure 1:**
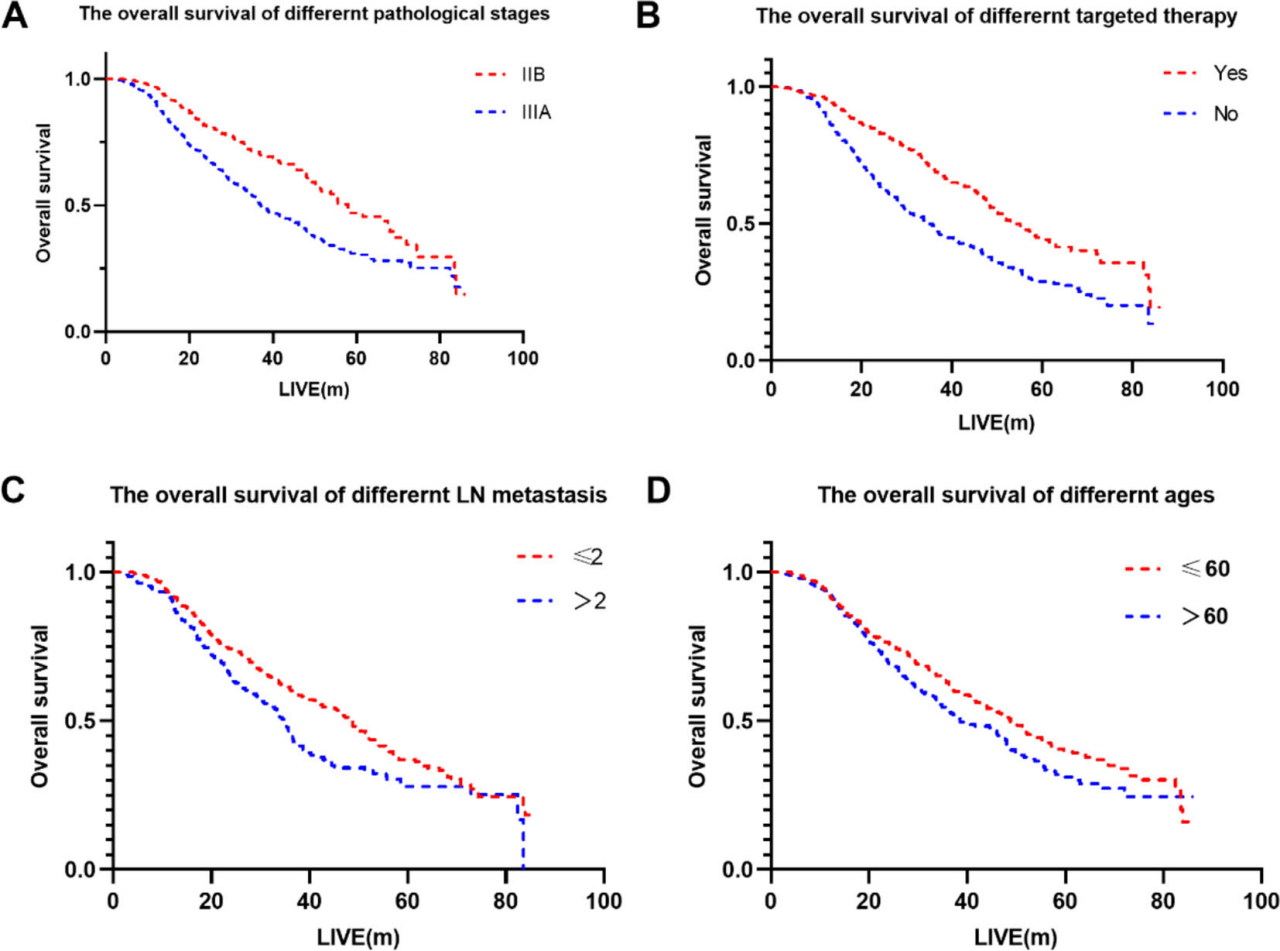
The overall survival of different factors. **(A)** The overall survival of different pathological stages. **(B)** The overall survival of different targeted therapy. **(C)** The overall survival of different LN metastasis. **(D)** The overall survival of different ages.

Univariate analysis identified gender, age, pathological stage, number of positive lymph nodes, postoperative chemotherapy and targeted therapy as the significant factors affecting OS (p < 0.1) (**Table 3**). Further analysis using multivariate Cox regression with the backward stepwise (Wald) method confirmed targeted therapy, pathological stage, age and the number of positive lymph nodes as independent prognostic factors associated with patients’ OS. The Kaplan-Meier survival curves for the independent risk factors are shown in **Figure 1**.

**Table 3:** Univariate and multivariate analyses of clinicopathological parameters for OS.

Factor	Univariate analysis		Multivariate analysis	
	P	HR (95%CL)	P	HR (95%CL)
**Age** >60 ≤60	**0.034**	1.245(1.015-1.526)	**0.047**	1.230(1.003-1.508)
**LN Metastasis** >2 ≤2	**<0.001**	1.474(1.186-1.843)	**0.022**	1.314(1.040-1.658)
**Pathological stage** IIIa IIb	**<0.001**	1.684(1.337-2.120)	**<0.001**	1.629(1.272-2.088)
**Gender** Male Female	**0.011**	1.299(1.060-1.590)	0.137	1.170(0.951-1.439)
**Surgical Method** Open VATS	0.158	1.252(0.916-1.712)		
**Air dissemination** Yes No	0.113	0.857(0.699-1.049)		
**Vascular invasion** Yes No	0.935	0.992(0.810-1.214)		
**Chemotherapy** Yes No	**0.013**	1.324(1.060-1.655)	0.613	1.065(0.835-1.357)
**Targeted therapy** Yes No	**<0.001**	0.565 (0.458-0.696)	**<0.001**	0.531(0.430-0.655)
**Radiotherapy** Yes No	0.232	0.861(0.674-1.101)		
**Immunotherapy**				
Yes	0.303	1.161(0.874-1.543)		
No				

Bold means *P* < 0.001 was considered very significant.

### Sequencing analysis and gene mutations

#### Patient baseline characteristics

A subset of 184 patients with unsuccessful postoperative targeted therapy and preserved tumor tissue was selected for whole-exome sequencing (WES). **Table 4** summarizes the clinical characteristics of these patients, showing that 56.0% were male, 51.6% were over 60 years old, 62.5% were had stage IIIa disease, 25.0% of the patients had >2 metastatic lymph node stations, approximately half of the patients had air dissemination or vascular invasion, and adjuvant chemotherapy was administered to 82.1%, while 22.3% received radiotherapy and 22.8% immunotherapy.

**Table 4:** The demographic and clinicopathological data of the patients.

Characteristics	Patients (n=184)
Gender	
Male	103 (56.0%)
Female	81 (44.0%)
Age	
>60	95 (51.6%)
≤ 60	89 (48.4%
LN metastasis	
>2	46 (25.0%)
≤ 2	138 (75.0%)
Chemotherapy	
Yes	151 (82.1%)
No	33 (17.9%)
Radiotherapy	
Yes	41 (22.3%)
No	143 (77.7%)
Immunotherapy	
Yes	42 (22.8%)
No	142 (77.2%)
Surgical Method	
Open	17 (9.2%)
VATS	167 (90.8%)
Air dissemination	
Yes	91 (49.5%)
No	93 (50.5%)
Vascular invasion	
Yes	90 (48.9%)
No	94 (51.1%)
Pathological stage	
IIIA	115 (62.5%)
IIB	69 (37.5%)

### The high-frequency mutated genes

**Figure 2:**
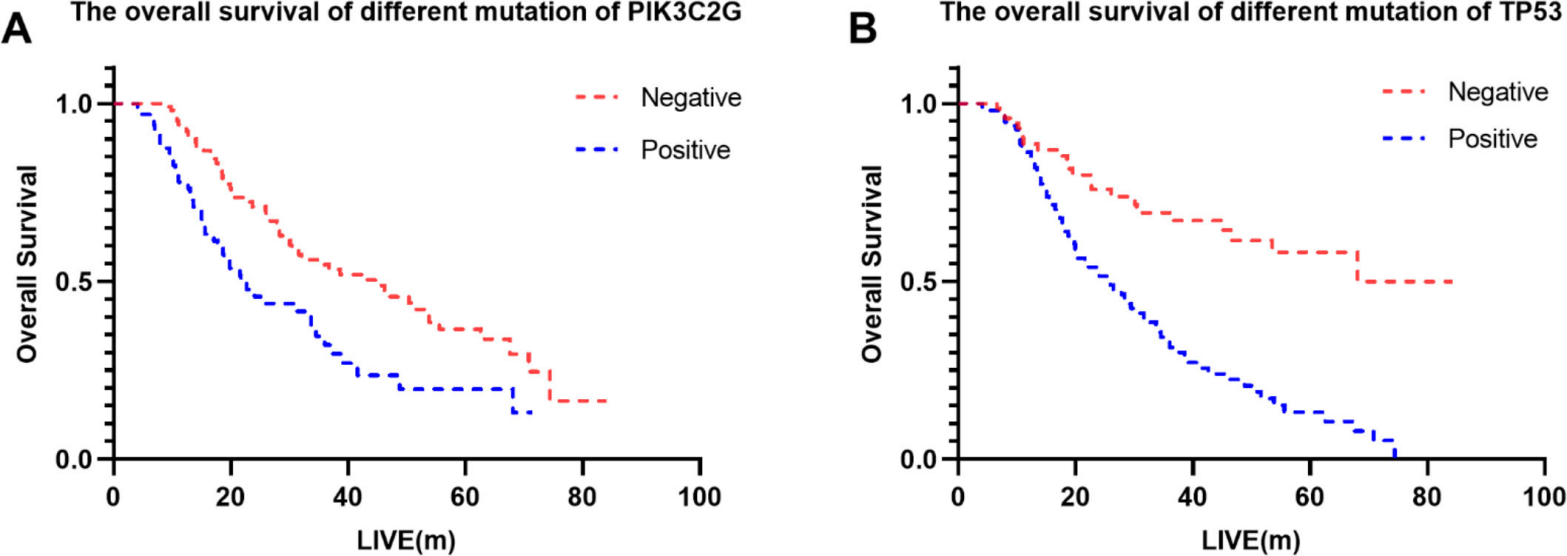
The overall survival of different mutation of **(B)** TP53 and **(A)** PIK3C2G.

WES identified several high-frequency mutated genes visualized using the “maftools” R package. **Supplementary Figure S1** shows the distribution of genes with mutation rates above 20%, including PIK3C2G (38.38%), TP53 (51.89%), and VEGFA (52.97%). Using Lasso regression (**Supplementary Figure S2**) and Cox regression models (**Table 5**), TP53 and PIK3C2G were identified as significant prognostic markers. The Kaplan-Meier survival curves for these genes are presented in **Figure 2**.

**Table 5:** Univariate and multivariate analyses of gene mutations.

Factor	Univariate analysis		Multivariate analysis	
	P	HR (95%CL)	P	HR (95%CL)
**VEGFA** + -	0.673	1.091 (0.727-1.638)		
**TP53** + -	**<0.001**	**3.175 (1.972-5.102)**	**<0.001**	**2.994 (1.852-4.831)**
**XPC** + -	0.577	0.890 (0.592-1.339)		
**KDR** **+** -	0.430	1.178 (0.785-1.767)		
**PIK3C2G** + -	**0.004**	**1.842 (1.220-2.778)**	**0.027**	**1.600 (1.055-2.427)**
**SLIT1** + -	0.782	1.062 (0.696-1.618)		
**CCND1** + -	0.289	0.815 (0.499-1.230)		
**CYP3A4** + -	0.733	0.912 (0.539-1.543)		
**XPA** + -	0.225	0.716 (0.418-1.227)		

Bold means *P* < 0.001 was considered very significant.

### Functional validation of PIK3C2G

Although numerous studies have been performed on TP53 and it is well-studied in lung cancer, the role of PIK3C2G remains unclear. To explore this, siRNA-mediated knockdown of PIK3C2G was performed in A549 cells alongside LTK, a known oncogenic driver in NSCLC, as a positive control. Functional assays were conducted to assess the effects on tumor progression, including cell proliferation, migration, invasion, apoptosis, and cell cycle distribution.

### Effective knockdown of PIK3C2G and LTK

RT-qPCR analysis confirmed the efficient silencing of PIK3C2G and LTK in A549 cells using targeted siRNAs (**Supplementary Figure S3**). Among the three siRNA candidates for each gene, the third siRNA demonstrated the most significant reduction in mRNA expression levels (p < 0.001), and was therefore selected for subsequent functional experiments.

### Reduced cell proliferation following PIK3C2G and LTK silencing

**Figure 3:**
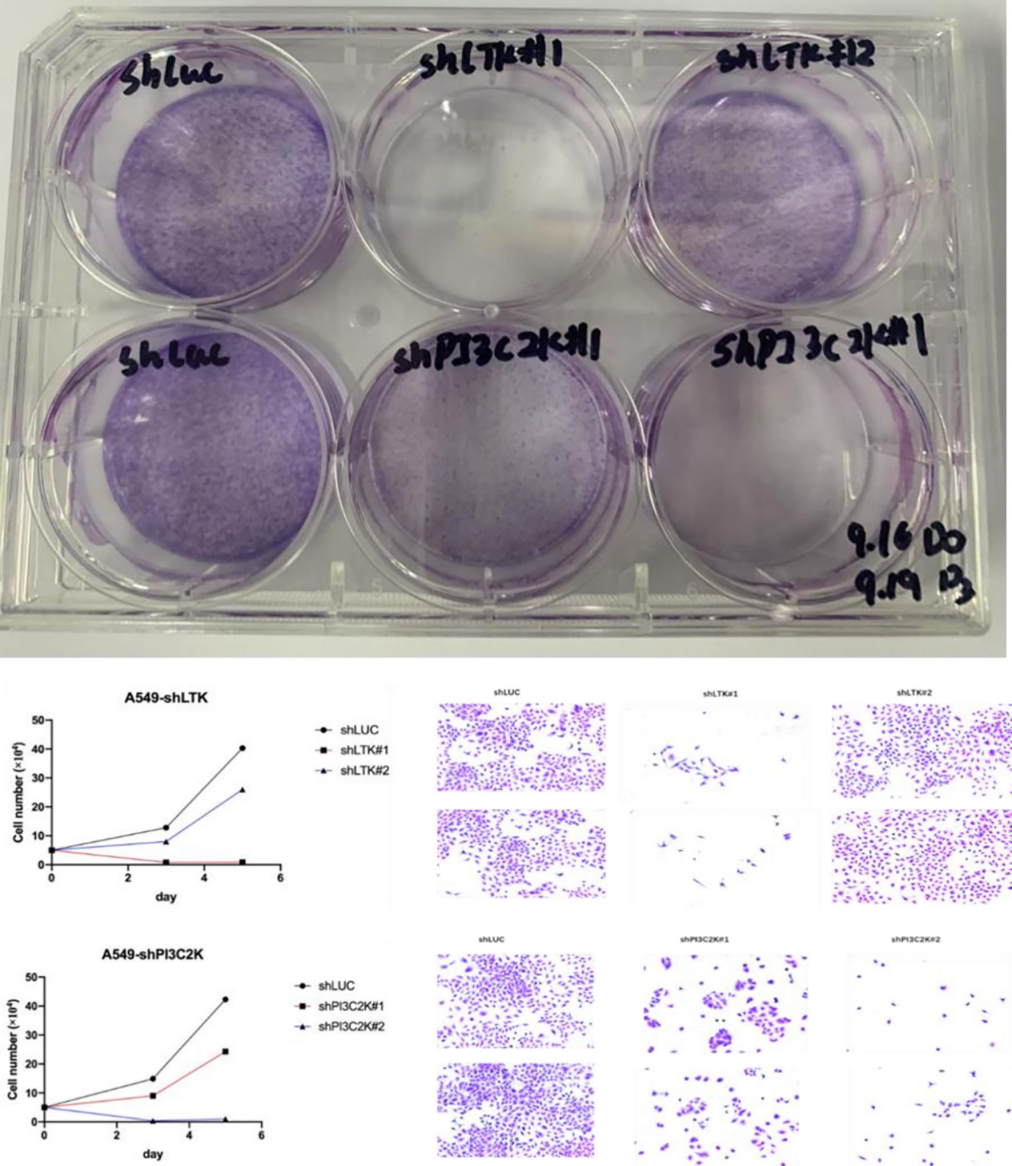
Cell proliferation significantly decreased after knocking down LTK and PI3C2K genes.

The knockdown of PIK3C2G and LTK significantly inhibited the proliferation of A549 cells over a 6-day culture period. As shown in **Figure 3**, cells transfected with siRNAs targeting either PIK3C2G or LTK exhibited markedly reduced growth rates compared to the control group, suggesting that both genes could play important roles in supporting cell proliferation in lung adenocarcinoma.

### Impaired migration and invasion of A549 cells

**Figure 4:**
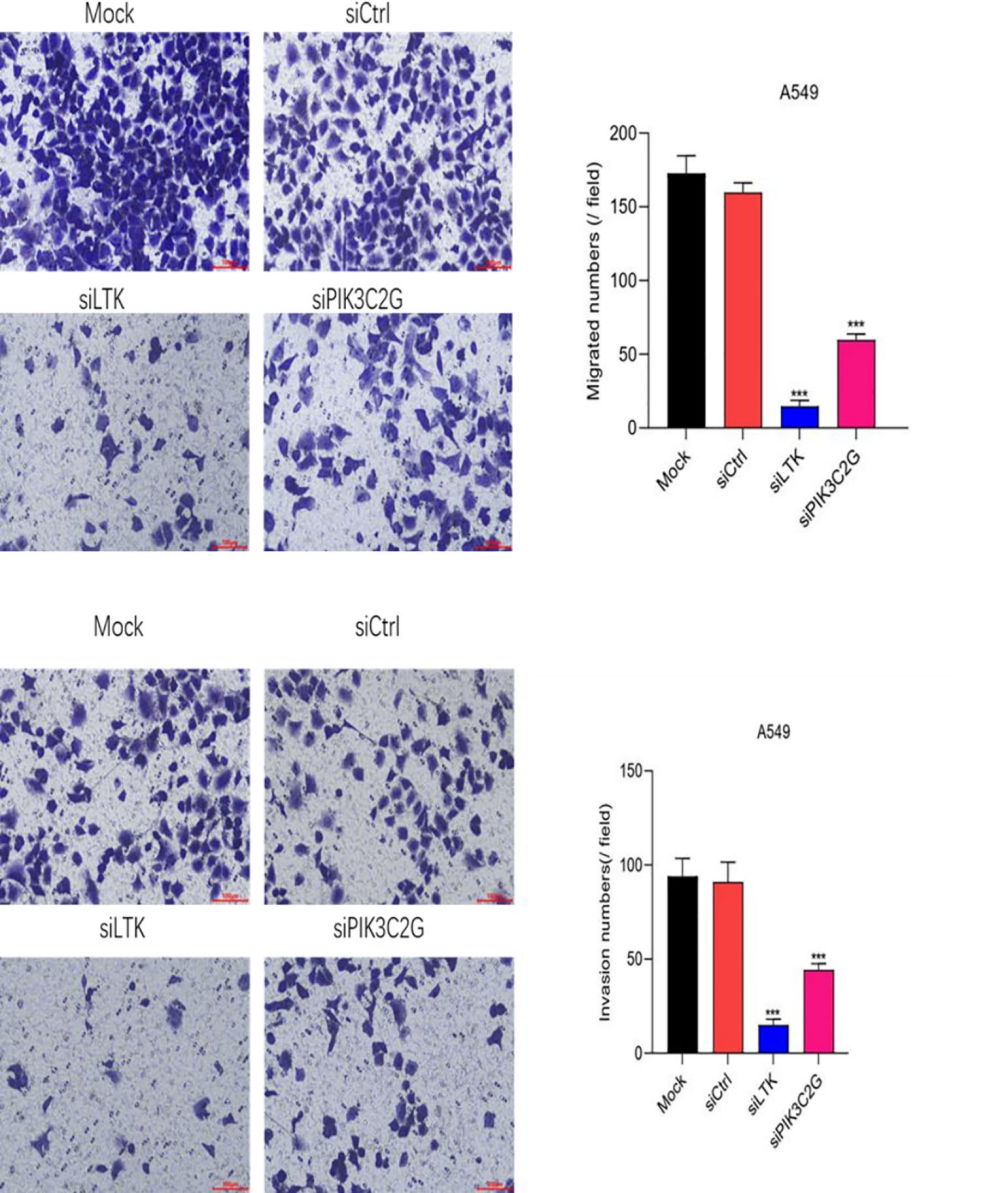
Cell migration and invasion abilities decrease after knocking down LTK and PIK3C2G genes. (Mock represents the blank control group, siCtrl is the internal control group with no gene silenced, and si-LTK and si-PIK3C2G represent the results of knocking down the respective genes. *** means P <0.001).

Transwell assays demonstrated that silencing PIK3C2G and LTK significantly reduced the migration and invasion capabilities of A549 cells (p < 0.001) (**Figure 4**), due to decrease in the number of cells traversing the membrane after gene knockdown. Indicating that both PIK3C2G and LTK contribute to the metastatic potential of lung adenocarcinoma cells.

### Enhanced apoptosis after PIK3C2G and LTK knockdown

**Figure 5:**
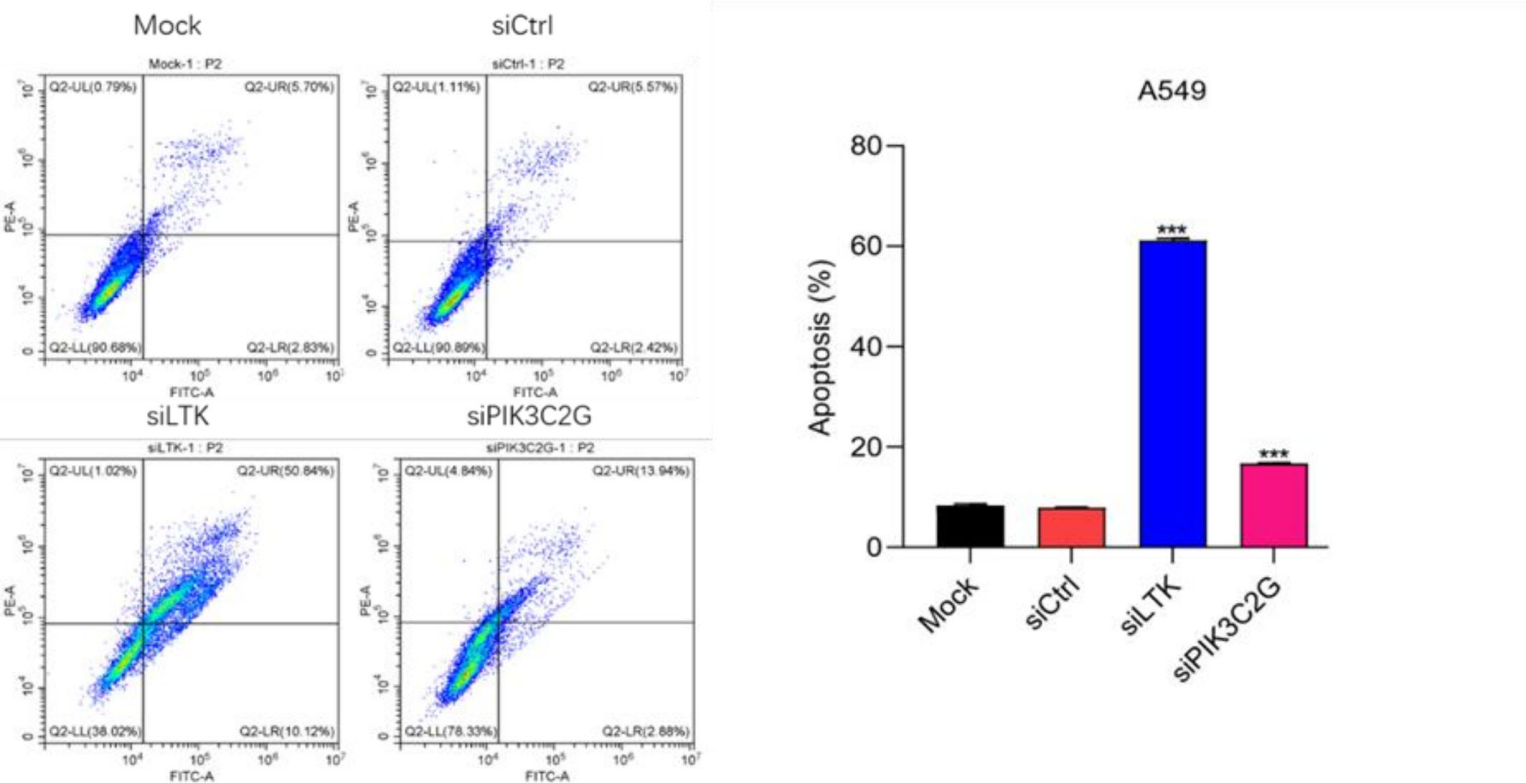
Knocking down LTK and PIK3C2G significantly promotes cell apoptosis. (Mock represents the blank control group, siCtrl is the internal control group with no gene silenced, and si-LTK and si-PIK3C2G represent the results of knocking down the respective genes. *** means P <0.001).

Flow cytometry analysis revealed a substantial increase in both early and late apoptotic A549 cells after silencing PIK3C2G and LTK compared to controls (p < 0.001) (**Figure 5**). These results indicate that PIK3C2G and LTK suppress apoptotic pathways, thereby promoting cell survival in lung adenocarcinoma.

### Disrupted cell cycle progression following knockdown

**Figure 6:**
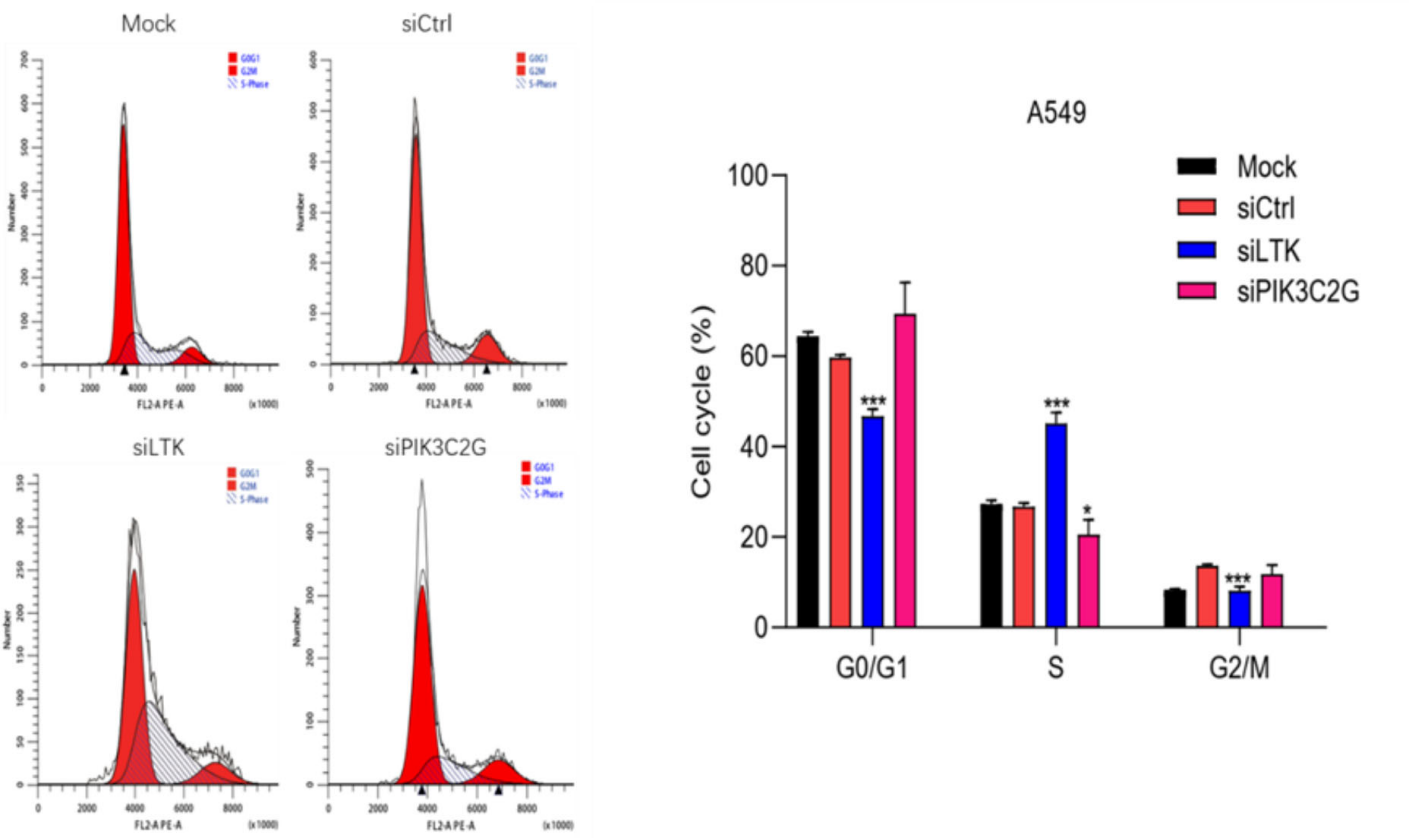
Knocking down the expression of LTK and PIK3C2G significantly promotes cell cycle progression. (Mock represents the blank control group, siCtrl is the internal control group with no gene silenced, and si-LTK and si-PIK3C2G represent the results of knocking down the respective genes. *** means P <0.001, * means P <0.1).

Gene silencing disrupted normal cell cycle progression in A549 cells (**Figure 6**). The knockdown of PIK3C2G caused cell cycle arrest in the G0/G1 phase, while LTK silencing resulted in an accumulation of cells in the S phase. These distinct effects suggest that PIK3C2G and LTK regulate different aspects of cell cycle progression, highlighting their unique roles in lung adenocarcinoma biology.

## Discussion

Lung cancer, due to its typically asymptomatic onset, more than 60% of patients are diagnosed at an advanced stage, contributing to a poor overall prognosis (7). Herein, we revealed that postoperative targeted therapy, pathological stage, age and the number of metastatic lymph node stations are key prognostic factors for stage IIb and IIIa lung adenocarcinoma, with advanced stage and greater lymph node involvement predicting worse outcomes. Whole-exome sequencing identified PIK3C2G as a novel prognostic marker alongside TP53. Functional validation in A549 cells revealed that PIK3C2G knockdown suppressed proliferation, migration, and invasion while promoting apoptosis and disrupting cell cycle progression, thus supporting PIK3C2G as a potential therapeutic target, warranting further exploration of its role in lung adenocarcinoma.

Age >60 has been shown to be a significant independent risk factor affecting lung cancer patient prognosis, due to associations with biological factors such as organ aging and decreased immune function, which can exacerbate tumor recurrence and metastasis in elderly patients (8, 9). Moreover, reduced tolerance to postoperative therapies, such as chemotherapy and immunotherapy, is common in older patients and can lead to poorer adherence to anti-cancer treatment regimens and contribute to reduced overall survival (10). These findings highlight the importance of tailoring treatment strategies for elderly patients, emphasizing the need for less aggressive yet effective regimens that balance treatment efficacy with quality of life.

The impact of gender on the prognosis of lung cancer patients remains controversial and varies across studies. It has been suggested that women with NSCLC generally have better prognoses than men of similar age and disease stage. Sachs et al. observed in a cohort of 6356 lung cancer patients that women undergoing lung cancer surgery had significantly better outcomes than men, with a hazard ratio of 0.73 (95% CI [0.67–0.79]) (11). Similarly, Cerfolio et al. reported that women demonstrated superior prognoses compared to men, particularly in the context of neoadjuvant chemotherapy, where women exhibited a higher objective response rate (79%) than men (51%, p = 0.025) (12). However, some studies have concluded that gender does not independently affect lung cancer survival outcomes (13). In our present study, gender was identified as a significant factor in univariate analysis but did not retain its significance in multivariate Cox regression, suggesting that while gender may influence lung cancer prognosis, it is likely confounded by other clinical and biological factors. Understanding the mechanisms underlying gender-related differences in prognosis could have substantial clinical implications, and further research is needed to elucidate the molecular and systemic factors contributing to these observations for improved outcomes across diverse patient populations.

Although pathological staging is an important independent prognostic factor for lung cancer, the prognostic significance of the number of metastatic lymph nodes has often been overlooked in clinical practice. The current N staging system categorizes lymph node involvement based on the anatomical location of metastases, and while this system effectively captures the extent of disease progression (14), it fails to incorporate the prognostic impact of the number of positive lymph node stations, which can provide additional insights into disease severity. Our study identified the number of metastatic lymph node stations as an independent risk factor for survival, with a higher number of affected stations correlating with worse outcomes. This finding aligns with the work of Misthos et al., who reported that patients with metastases in ≥2 mediastinal lymph node stations experienced significantly poorer prognoses (15), underscoring the importance of revisiting the current N staging. Incorporating the number of positive lymph node stations into clinical decision-making could enhance the precision of prognostic assessments, guide the stratification of patients for adjuvant therapy, and inform postoperative follow-up strategies, ultimately improving personalized treatment planning and optimizing lung cancer patients outcomes.

Adjuvant chemotherapy with cytotoxic drugs theoretically benefits lung cancer patients by reducing tumor burden and targeting micrometastases, with platinum-based regimens recommended for stage II–IIIA disease to lower the absolute risk of death by 5.4% within five years (16). However, our findings showed no significant improvement in prognosis, potentially due to factors such as limited sample size, variability in chemotherapy regimens, and the concurrent use of other treatments such as targeted therapy, radiotherapy and immunotherapy. Targeted therapies have demonstrated superior outcomes for patients with specific genetic mutations, such as EGFR (17), ALK (18), and ROS1 (19), RET (20), BRAF V600E (21), MET Exon 14 (20), and NTRK (21), significantly improving survival and reducing recurrence risks. For instance, osimertinib for EGFR mutations and alectinib for ALK rearrangements can extend progression-free survival and reduce CNS progression. In this study, targeted therapy emerged as the only protective prognostic factor, emphasizing the essential role of genetic testing in guiding treatment for stage IIb–IIIa lung adenocarcinoma. Nevertheless, the limited availability of actionable mutations leaves a substantial proportion of patients without targeted therapy options, underscoring the need to identify new therapeutic targets. In this regard, our analysis identified TP53 and PIK3C2G as potential prognostic markers, providing a basis for further research into personalized therapeutic strategies for these patients.

Exons, the protein-coding regions of the human genome, constitute only about 1% of the genome but harbor approximately 85% of pathogenic mutations (22). This concentration of clinically relevant mutations makes exome sequencing a highly efficient approach for genomic analysis, especially in the context of tumor heterogeneity. Tumors often comprise numerous subclones, including those with low representation, necessitating high-depth sequencing to identify low-frequency and rare variants effectively (23). WES offers significant advantages over whole-genome sequencing (WGS) by allowing deeper sequencing coverage of exons, thereby enhancing the detection of rare mutations while reducing costs and data storage requirements (24), making WES particularly valuable for identifying key driver mutations in solid tumors (25).

The LTK gene encodes leukocyte receptor tyrosine kinase, a member of the tyrosine kinase family involved in regulating cell growth and differentiation. With multiple transcript variants encoding distinct isoforms, LTK plays a complex and critical role in cellular processes. Izumi et al. identified the CLIP1-LTK fusion protein as a lung cancer driver, accounting for 0.4% of cases, and demonstrated that LTK rearrangements, identified using fluorescent probes targeting the 5’ and 3’ ends of LTK, were present in 18% of cancer cells, forming the CLIP1-LTK fusion protein. They further showed that treating CLIP1-LTK-positive lung cancer cells with the ALK inhibitor lorlatinib effectively inhibited kinase activity, suppressed proliferation, and induced apoptosis (26). In our present study, LTK knockdown was used as a positive control, providing a reliable reference for assessing its role in lung cancer progression and for comparative evaluation of PIK3C2G’s functional impact in A549 cells.

The PI3K-AKT-mTOR pathway is central to tumor-related processes such as cell growth, survival, and progression, with somatic mutations frequently observed in various cancers. While PIK3CA mutations are strongly associated with lymph node metastasis and increased tumor invasiveness (27), PIK3C2G, another key PI3K family member, has emerged as a potential tumor suppressor. Studies in colorectal cancer have shown that low PIK3C2G copy numbers significantly increase recurrence and mortality risks (28), and in pancreatic cancer, PIK3C2G knockout in mouse models accelerated tumor progression, reduced survival, and increased drug resistance by disrupting mTORC1-mediated pathways (29). Beyond these malignancies, targeting PI3K signaling, including PIK3C2G, has shown therapeutic promise in cancers such as hepatocellular carcinoma, breast cancer, and renal cell carcinoma (30–33). These findings highlight the need to further investigate PIK3C2G’s role in lung adenocarcinoma, particularly as a potential biomarker or therapeutic target to enhance personalized treatment strategies.

Evidence suggests that PIK3C2G and its related gene PIK3CG contribute to tumor progression and metastasis. Mutations in PIK3CG have been associated with shorter survival in squamous cell lung cancer and increased mutation frequencies in brain metastases compared to primary tumors (34–38). Experimental models, including lung metastasis studies, indicate that PIK3CG enhances metastatic colonization, cell survival, and malignant proliferation (39). Similarly, our findings demonstrate that knocking down PIK3C2G in A549 cells significantly inhibits proliferation, migration, and invasion while increasing apoptosis and disrupting cell cycle progression. These effects align with Sun J’s observations that PIK3CG promotes lung cancer metastasis through mechanisms such as matrix metalloproteinase expression and neutrophil-mediated tumor microenvironment changes (40). These findings suggest that targeting PIK3C2G could suppress lung cancer growth and metastasis, offering a promising avenue for therapeutic development to improve treatment outcomes and patient quality of life.

This study has several limitations that should be addressed. First, as a single-center retrospective study with a relatively small sample size, its findings may not fully represent the general population. Second, postoperative treatments were broadly categorized into chemotherapy, targeted therapy, radiotherapy and immunotherapy without detailed stratification of specific regimens, treatment courses, or associated adverse reactions, which might have introduced bias in the analysis. Third, while the prognostic role of PIK3C2G was explored, external validation of these findings was lacking, and experimental studies were limited to the cellular level without protein-level investigations. Future research could aim to incorporate larger, multi-center cohorts, provide more detailed stratification of treatment protocols, and validate PIK3C2G’s role through comprehensive experimental studies, including protein-level analyses.

## Conclusion

This study provides a comprehensive analysis of postoperative survival in stage IIb and IIIa lung adenocarcinoma patients, identifying key clinical and pathological factors that influence prognosis, which could be considered to develop personalized treatment strategies. WES identified PIK3C2G as a novel prognostic marker alongside the well-established TP53 gene. Functional experiments demonstrated that silencing PIK3C2G inhibits cell proliferation, migration and invasion, while promoting apoptosis and altering cell cycle progression in A549 cells. These findings suggest that PIK3C2G may serve as a promising therapeutic target, emphasizing the importance of further validation and exploration of its role in lung adenocarcinoma to improve clinical outcomes.

## Data Availability

The raw data supporting the conclusions of this article will be made available by the authors, without undue reservation.
