# Supplementary figures and images for: Prognostic determinants and functional role of PIK3C2G in stage IIb-IIIa lung adenocarcinoma: insights from clinical and molecular analyses
Source: Front Oncol. 2025 Jan 30;14:1473437. doi: 10.3389/fonc.2024.1473437 (PMC11821497; doi:10.3389/fonc.2024.1473437)

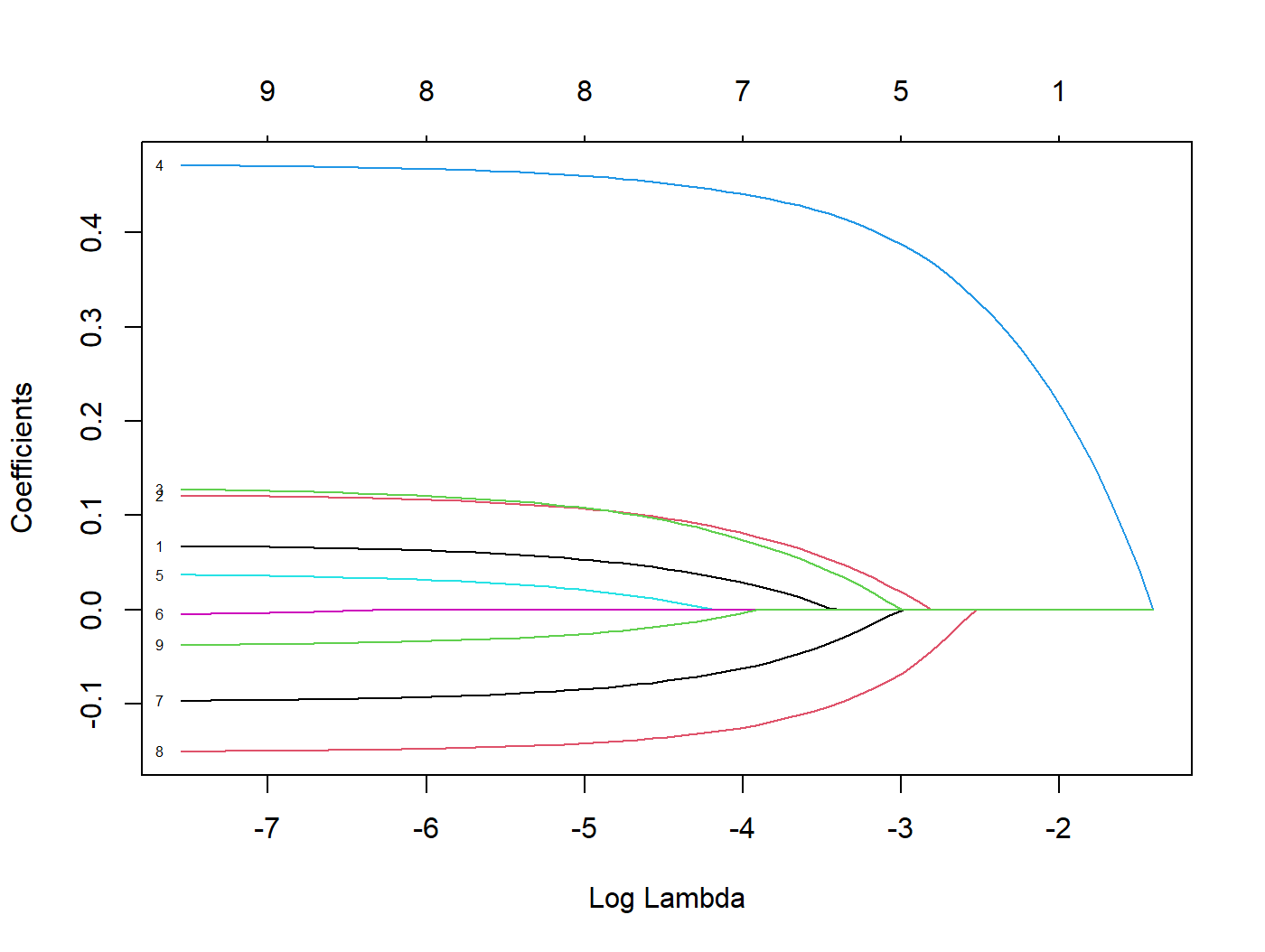

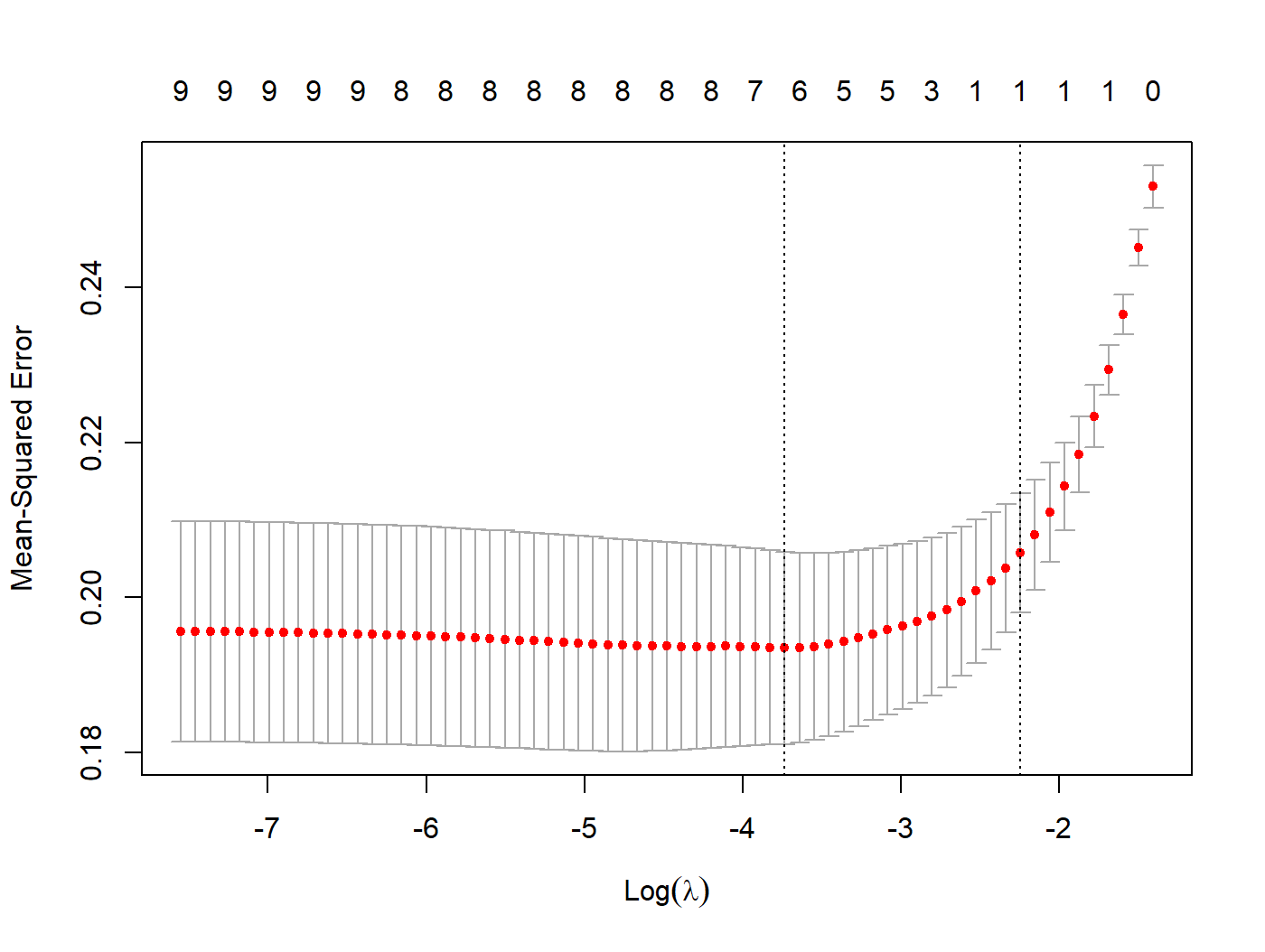

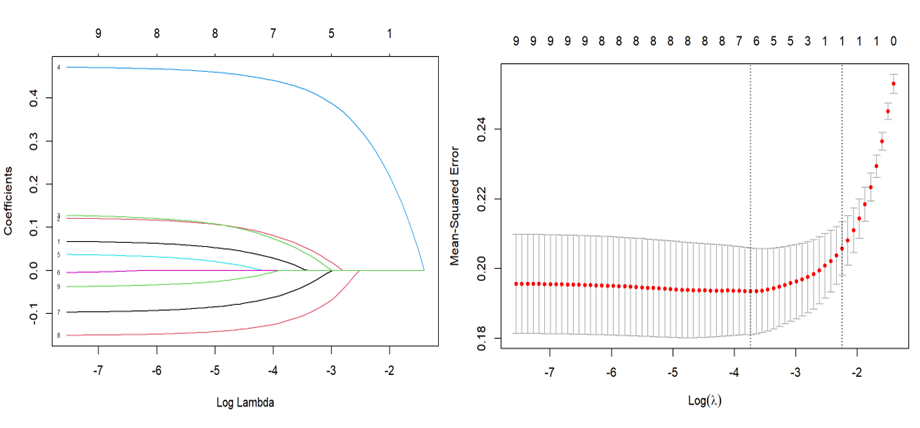


**Supplement 2.** The result of Lasso regression

Supplement: Supplementary file 2 [file DataSheet2.docx]

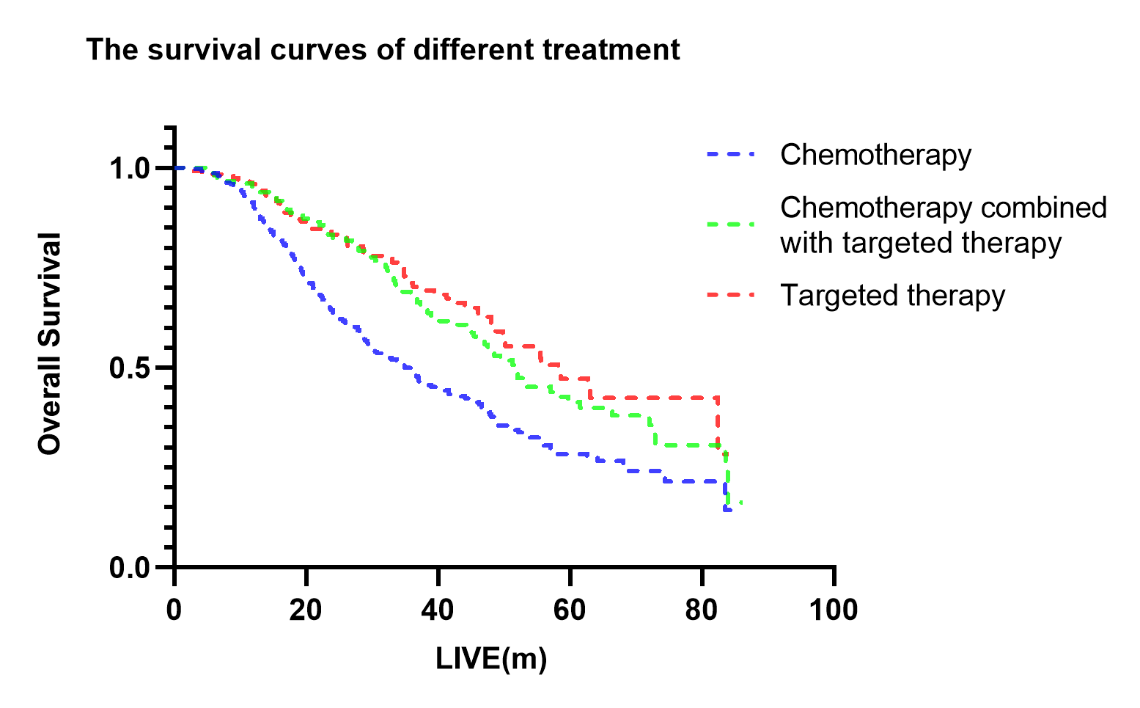


**Supplement 4.** The survival curves of different treatment

Supplement: Supplementary file 4 [file DataSheet4.docx]
