# Supplementary material for: Prognostic determinants and functional role of PIK3C2G in stage IIb-IIIa lung adenocarcinoma: insights from clinical and molecular analyses
Source: Front Oncol. 2025 Jan 30;14:1473437. doi: 10.3389/fonc.2024.1473437 (PMC11821497; doi:10.3389/fonc.2024.1473437)
Supplement: Supplementary file 3 [file DataSheet3.docx]

**Supplement 3.** PCR Detection Results Following Transfection of A549 Cells with Three siRNAs Targeting PIK3C2G and LTK (*** means P <0.001)
