# Supplementary material for: Prognostic determinants and functional role of PIK3C2G in stage IIb-IIIa lung adenocarcinoma: insights from clinical and molecular analyses
Source: Front Oncol. 2025 Jan 30;14:1473437. doi: 10.3389/fonc.2024.1473437 (PMC11821497; doi:10.3389/fonc.2024.1473437)
Supplement: Supplementary file 5 [file Table1.docx]

**Supplementary Table S1. qPCR primer sequences**

| Gene | | qPCR primer sequences |
| --- | --- | --- |
| LTK | F： | TTCCCGGTCACATCGAGAG |
|  | R： | TCCTGTTGCATACCGTCTAAATC |
| PIK3C2G | F： | TCAGGAAGATATATGCGTCAGGAAA |
|  | R： | AGAGGGTTGACTCTGCTCCA |
| β-actin | F： | CATGTACGTTGCTATCCAGGC |
|  | R： | CTCCTTAATGTCACGCACGAT |
